# Image‐based robotic total knee arthroplasty preserves the knee joint line level even in advanced fixed flexion deformities when combined with functional alignment principles: A retrospective comparative cohort study

**DOI:** 10.1002/ksa.12643

**Published:** 2025-03-07

**Authors:** Giacomo Pacchiarotti, Alessandro Todesca, George Mihai Avram, Giovanni Longo, Domenico Paolicelli, Stefano Gumina

**Affiliations:** ^1^ Department of Anatomy, Histology, Legal Medicine, and Orthopaedics Sapienza University of Rome Rome Italy; ^2^ Istituto Chirurgico Ortopedico Traumatologico (ICOT) Latina Italy; ^3^ Department of Orthopedic Surgery and Traumatology Kantonsspital Baselland Bruderholz Switzerland

**Keywords:** fixed flexiondeformity, functional alignment, image‐based, knee joint line, robotic‐assisted surgery, total knee arthroplasty

## Abstract

**Purpose:**

Fixed flexion deformity (FFD) is traditionally addressed in total knee arthroplasty (TKA) with extensive soft tissue release and distal femoral recut, which increases bone stock consumption and raises the knee joint line (JL). This study aimed to evaluate differences in the anatomical restoration of the JL and bone stock preservation between FFD knees and a control group during robotic‐assisted (RA) TKA combined with functional alignment (FA).

**Methods:**

A retrospective comparative cohort study examined 120 knees undergoing RA TKA. The knees were categorised into two groups: the study group, with FFD > 5°, and the control group, without FFD. Further analysis stratified the study group based on the severity of the deformity: mild (5–9°), intermediate (10–14°) and advanced (>15°). The Mann–Whitney *U* test was utilised to investigate the differences between the control and study groups.

**Results:**

The study group comprised 64 knees, presenting an average flexion contracture and range of motion (ROM) of 11.3 ± 4.7° and 112.7 ± 11.6°, respectively. The control group comprised 56 knees, with an extension deficit and ROM of 1.6 ± 2.1° and 123.5 ± 8.3°, respectively. The JL was proximally displaced on average by 0.1 ± 1.2 mm in the study group and lowered by 0.7 ± 0.9 mm in the control group. Analysis of JL in the subgroups showed a lowering of 0.3 ± 1.2 mm in the mild deformity subgroup and a rise of 0.08 ± 1.3 mm and 0.8 ± 0.8 mm in the intermediate and advanced FFD subgroups, respectively, showing no statistical significance. The combined thickness of tibial proximal and femoral distal bone cuts measured 12.3 ± 1.6 mm in the study group and 11.4 ± 1.4 mm in the control group.

**Conclusions:**

FA in RA‐assisted TKA can correct FFD, minimising bone cuts while preserving anatomical JL level.

**Level of Evidence:**

Level III.

**Abbreviations**
CR: cruciate‐retaining
FA: functional alignment
FFD: fixed flexion deformity
HKA: hip–knee–ankle
JL: joint line
KA: kinematic alignment
MA: mechanical alignment
mLDFA: mechanical lateral distal femoral angle
mMPTA: mechanical medial proximal tibial angle
OA: osteoarthritis
PCL: posterior cruciate ligament
PS: posterior‐stabilized
RA: robotic assistance
ROM: range of motion
TKA: total knee arthroplasty

## INTRODUCTION

Fixed flexion deformity (FFD) is characterised by the inability to extend the knee joint, neither actively nor passively. This condition is commonly associated with osteoarthritis (OA) and is typically identified when there is a contracture in flexion of at least 5° [15, 39]. The incidence of FFD in knee OA reaches approximately one‐third of patients, and its development is multifactorial, involving bone impingement, osteophytes, and several contractures affecting the posterior capsule, ligaments and tendons [5, 39, 47].

FFD is treated in conjunction with OA through total knee arthroplasty (TKA). This procedure traditionally employs a mechanical alignment (MA) technique, which aims to achieve a neutral alignment by positioning the prosthetic components parallel to each other and perpendicular to the mechanical axis of the limb [35]. This approach addresses flexion deformity through a combination of soft tissue releases and femoral re‐resections to increase the extension gap [2]. However, this strategy has several drawbacks. Soft tissue releases can alter proprioceptive function and balance, leading to altered joint kinematics, slower rehabilitation and reduced patient satisfaction [3]. Re‐resection of the distal femur elevates the joint line (JL), leading to mid‐flexion instability and potentially causing patellofemoral joint problems [23, 29].

Over the past decades, alternative solutions have been developed to minimise the need for soft tissue releases and recutting. Kinematic alignment (KA) was introduced to restore pre‐arthritic anatomy through manually measured bone resections, achieving good results and reducing the need for soft tissue release [41].

The subsequent introduction of robotic assistance (RA) has further improved precision and ease of use, proving to be a valuable tool in correcting deformities during TKA [28]. Robotic systems enable precise planning of limb alignment and component positioning in three‐dimensional (3D) space by virtually simulating joint gaps before performing bone cuts. These opportunities have led to the development of an anatomical‐functional alignment (FA) of the lower limb [36]. FA strategy addresses deformities by precisely positioning each component on every plane to restore proper joint gaps, ensures a full ROM and optimises patellofemoral tracking while minimising the necessity for ligament releases [7, 49, 50].

While this technology shows potential for treating flexion deformities by minimising JL displacement and bone loss, its effectiveness is still barely reported and consistently associated with KA principles [32, 42].

This retrospective analysis aimed to evaluate the outcomes of FA principles between patients with FFD and a control group, focusing on the anatomical reconstruction of the JL and the preservation of the epiphyseal bone stock during RA TKA.

## MATERIALS AND METHODS

### Ethical approval

The present study employs a non‐interventional/observational methodology. It thereby does not fall under the Italian Legislative Decree no. 211 of June 2003, a transposition of Directive 2001/20/EC relating to good clinical practice in clinical trials. Using an observational approach, the assignment of the patient to a particular procedure was not decided in advance according to a trial protocol but falls instead under current standards of clinical practice, and no additional diagnostic, therapeutic, or monitoring procedures were applied. Only epidemiological methods were used to analyse the collected data in a blinded form. All patients provided their consent by signing the ‘General Consent’ form, which included an agreement for the retrospective use of their data. Therefore, formal ethical approval was not required for this study.

### Study population

The present study was conducted according to the STROBE guidelines using a retrospective, single‐centre, comparative cohort design. In accordance with the principles of the Declaration of Helsinki and Good Clinical Practice guidelines, a retrospective review of the hospital's database was conducted. A total of 117 patients (120 knees) who underwent RA‐assisted TKA between February 2021 and January 2024 met the inclusion criteria. All procedures were performed using the MAKO® image‐based robotic‐assisted surgical system (Stryker) with either a cruciate‐retaining (CR) or posterior‐stabilized (PS) Triathlon implant (Stryker) (Table 1). Patients with clinical and radiographic signs of end‐stage OA (Kellgren–Lawrence Grades III and IV) were considered eligible regardless of the severity of coronal deformities and the presence of any degree of flexion deformity. Individuals who underwent previous knee osteotomies, partial knee replacements or were affected by OA secondary to traumatic, inflammatory or septic conditions, as well as patients affected by hyperextension over 5° or neurological diseases affecting the lower extremities, were excluded.

**Table 1:** Patient demographics data.

	Control group	Study group	*p* a
Patient distribution (*n*)	53	64	
Age at surgery (years old)	71 ± 6 (60–85)	72 ± 5 (58–86)	0.66
Body mass index (kg/m²)	27 ± 4 (23–35)	28 ± 3 (25–33)	0.23
Gender distribution (*n*)			
Male	26	45	
Female	27	19	
Side (%)			
Right	62.3 (*n* = 33)	45.3 (*n* = 29)	
Left	32 (*n* = 17)	54.7 (*n* = 35)	
Bilateral	5.7 (*n* = 3)	0 (*n* = 0)	
Alignment (%)			
Varus	47.2 (*n* = 25)	64 (*n* = 41)	
Valgus	35.8 (*n* = 19)	36 (*n* = 23)	
Neutral	17 (*n* = 9)		
Implant type (%)			
Cruciate‐retaining	69.7 (*n* = 39)	51.6 (*n* = 33)	
Posterior‐stabilized	30.3 (*n* = 17)	48.4 (*n* = 31)	

*Note*: Parametric data are expressed as mean values ± standard deviation and range.

^a^ Determined using the Mann–Whitney *U* test.

### Surgical technique

**Figure 1:**
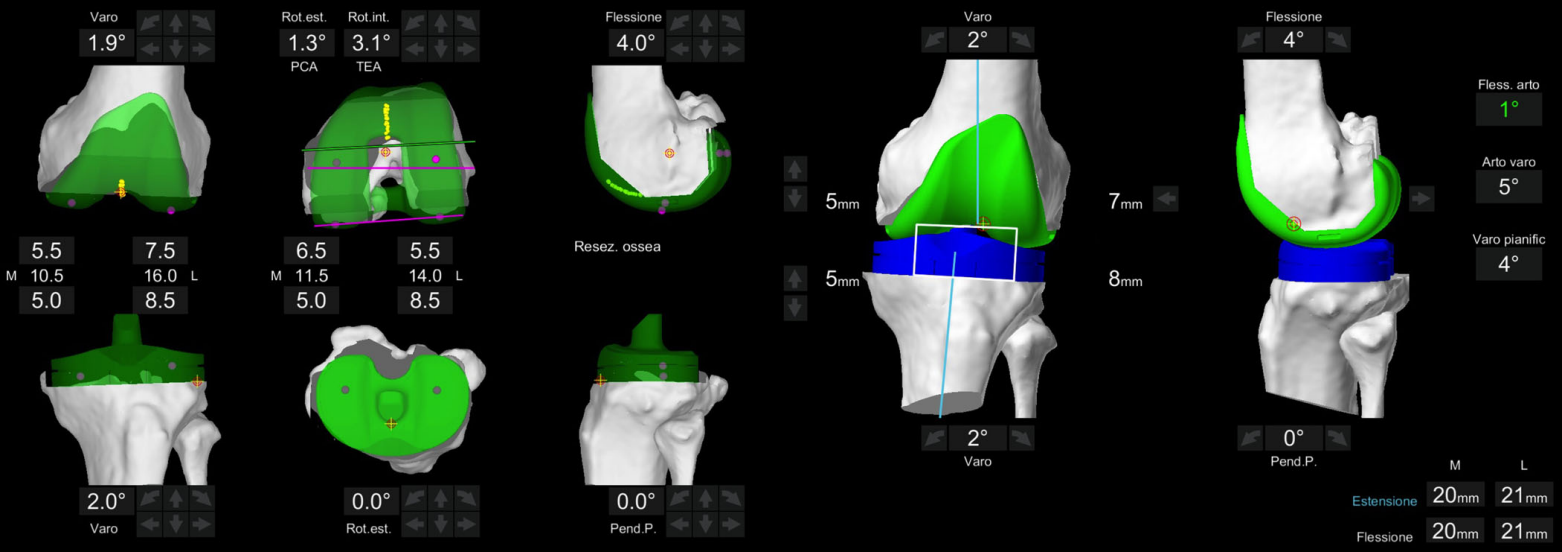
Intraoperative imaging demonstrates the alignment of knee components using functional alignment principles for a varus knee. The procedure corrected initial deformities of 5° varus and 21° fixed flexion deformity without needing ligament release. This approach achieved a final combined functional varus alignment of 4°, ensuring native joint line level and full knee extension.

Each surgical procedure was performed under spinal anaesthesia and peripherical nerve blocks. A midvastus approach with lateral dislocation of the patella was performed in each case. After joint exposure, optical motion‐capture trackers were positioned within the surgical field on both the femoral and tibial metaphyses [48]. This setup was applied to avoid interfering with patellar reduction or ROM assessment. A computed tomography (CT)‐based 3D model was prepared for each case and used for preoperative planning. A sharp optical tracker probe matched intraoperative bony landmarks to the 3D reconstructed model. Before menisci and osteophytes removal, ROM and limb alignment were collected. Flexion deformity was measured with the patella reduced and the leg supported by the heel without any force application. The posterior cruciate ligament (PCL) was tested during the knee preparation to determine the implant type. A CR implant was adopted for patients with an intact PCL. In contrast, a PS implant was chosen for cases involving a deficient PCL or when its integrity or strength was uncertain. After menisci and osteophytes removal, ligamentous tension was measured in flexion and extension to finalise the operative planning. After cartilage assessment with a blunt probe, limb alignment and JL alignment were personalised for each case to define the optimal degree of correction following the principles of FA [36, 44] (Figure 1). Considering the distal femoral cartilage is approximately 2 mm thick, preoperative CT planning was conducted for each patient, starting with a planned 6.5 mm distal femoral resection [24]. Intraoperatively, the resection thickness was tailored to each patient to restore their native pre‐arthritic alignment and preserve a natural functional ligament balance. The coronal alignment of the femur was set between 3° varus and 5° valgus relative to the mechanical lateral distal femoral angle (mLDFA), while tibial coronal positioning was planned between 5° varus and 3° valgus, matching the mechanical medial proximal tibial angle (mMPTA). Overall, the total mechanical deviation from the neutral hip–knee–ankle (HKA) angle never exceeded 5° in either varus or valgus from the neutral axis. The implant size was selected to achieve balanced extension and flexion gaps with 1–2 mm of asymmetrical lateral laxity in flexion, avoiding femoral notching and mediolateral overhang. The femoral component was positioned with 0–5° of flexion and a rotation ranging from 3° internal to 6° external relative to the surgical transepicondylar axis. These adjustments were customised to each patient's anatomy, specifically to align with the orientation and shape of the native trochlea following groove assessment with the blunt probe. The tibial component's size was determined by ensuring the best coverage of the tibial epiphysis without causing medial overhang. If needed, flexion space was addressed with slope correction between 0° and 3°. After the planning stage, component sizing was double‐checked on their margins using the blunt probe; then, bone cuts were performed with the assistance of the haptic robotic system. Careful removal of the posterior osteophytes was performed with trial components in place. MAKO optical trackers in the surgical field were used to measure maximum extension, full ROM and coronal laxity of the knee, employing the techniques previously described for the pre‐resection data. Medial soft tissue releases were performed in three cases with associated severe varus coronal deformity before intraoperative planning. Bone recuts were not necessary in any of the cases. Each knee was balanced using a minimum insert thickness of 9 or 11 mm, except for one case where a 13‐mm insert was required due to significant bone loss from severe varus deformity. The arrays were removed, and the epiphyses were prepared for cementation and components implanted using the standard cementation technique.

### Data collection

Demographic data were collected from the patient's medical record. Intraoperative data were collected directly from the robotic platform with an accuracy of within 1 mm [45]. Femoral distal and tibial proximal coronal bone resection thickness, full ROM, including maximum flexion and extension angles, along with lower limb alignment parameters such as mLDFA, mMPTA and HKA angle, have been recorded intraoperatively before and after bone cuts and trial implant placement. Distal femoral cuts have been double‐checked through a mechanical calliper with an accuracy of 1 mm. The thickness of the epiphyseal resections of the distal femur plus 2 mm of cartilage was combined with the 8.5 mm thickness of the implant, a single radius femoral component (Triathlon®, Stryker), to calculate the displacement of the JL, expressed as the femoral articular surface line. Coronal total tibial and femoral resections were combined to determine the total resected bone. The patients were divided into two groups: the first group included knees with FFD greater than 5° (study group), and the second group included knees with physiological extension (control group). Further analysis involved stratifying the study group into three subgroups based on the degree of flexion deformity [5]: mild, from 5° to 9°; intermediate, from 10° to 14°; and advanced deformity, over 15°.

### Data analyses

Collected data were tabulated in a Microsoft Excel® sheet (Microsoft Corporation) and analysed using SPSS software, version 22.0 (IBM‐SPSS). The correction coefficient was not calculated as we extrapolated the data from the robotic navigation system. Descriptive statistical analysis was reported with mean, median, range and standard deviation values with 95% confidence interval (CI). We performed a Shapiro–Wilk test to evaluate the normality of the distribution. Following this, the Mann–Whitney *U* test for two unrelated samples was used to compare items from the study and control groups before and after knee replacement. All statistical tests were performed two‐sided. Statistical significance was considered at *p* value < 0.05 for all analyses.

## RESULTS

The research involved 117 patients, including 71 men and 46 women, distributed as 62 right knees, 52 left knees and three bilateral cases for 120 included knees. During the preoperative assessment, the study group included 64 knees, 41 in varus and 23 in valgus alignment, presenting a mean HKA of 173.9 ± 6.2° (95% CI: 171.7–176.2). The average extension deficit was 11.3 ± 4.7° (95% CI: 9.7–13), with a full ROM measuring 112.7 ± 11.6° (95% CI: 108.6–116.8). The control group comprised 56 knees, including 27 varus, 20 valgus and nine neutral alignments, with a mean HKA of 179.2 ± 7.4° (95% CI: 176.2–182.2). The extension was deficient on average of 1.6 ± 2.1° (95% CI: 0.8–2.4), while total ROM averaged 123.5 ± 8.2° (95% CI: 120.1–126.8). Demographic data are detailed in Table 1.

Post‐operative analysis reported comparable results regarding HKA, ROM and maximum extension across the two groups (Table 2). Mean post‐operative HKA was, on average, 177.8 ± 2.1° (95% CI: 177–178.5), along with an average full ROM of 134.3 ± 5.2° (95% CI: 132.4–136.2) and a maximum extension of −0.3 ± 2.2° (95% CI: −1.1 to 0.4) in the study group. Mean HKA was restored at 178.9 ± 2.4° (95% CI: 178–179.9), and total ROM reached an average of 134.9 ± 4.8° (95% CI: 133–136.9) with a maximum extension of −0.9 ± 1.9° (95% CI: −0.2 to −1.7) in the control group (Table 2).

**Table 2:** Comparison of mean perioperative joint replacement outcomes and epiphyseal resection results between patients with fixed flexion deformity and those in the control group.

	Control group, *N* = 56	Study group (>5°), *N* = 64	*p* a
Distal femoral resection (mm)	5.75 ± 0.87	6.61 ± 1.20	0.19
Proximal tibial resection (mm)	5.63 ± 1.07	5.73 ± 1.19	0.97
Total bone resected (mm)	11.38 ± 1.39	12.34 ± 1.56	0.13
JL displacement (mm)	−0.75 ± 0.86	0.11 ± 1.20	0.25
Preoperative			
Extension deficit (°)	1.59 ± 2.10	11.39 ± 4.74	0.007*
Maximum flexion (°)	125.077 ± 8.75	124.10 ± 8.33	0.83
Range of motion (°)	123.46 ± 8.30	112.70 ± 11.60	0.05*
HKA angle (°)	179.19 ± 7.41	173.97 ± 6.25	0.33
Post‐operative			
Extension deficit (°)	−0.92 ± 1.90	−0.34 ± 2.19	0.52
Maximum flexion (°)	134 ± 4.66	133.97 ± 4.44	0.98
Range of motion (°)	134.92 ± 4.80	134.31 ± 5.22	0.944
HKA angle (°)	178.95 ± 2.39	177.79 ± 2.13	0.39
mLDFA (°)	90.42 ± 1,73	90.89 ± 1.79	0.47
mMPTA (°)	89.61 ± 0.99	89.27 ± 0.91	0.59

*Note*: Data distribution is presented as mean values ± standard deviation and range.

Abbreviations: HKA, hip–knee–ankle; JL, joint line; mLDFA, mechanical lateral distal femoral angle; mMPTA, mechanical medial proximal tibial angle.

^a^ Differences between study and control groups before and after knee replacement (Mann–Whitney *U* test).

* Significant *p* value (<0.05).

**Figure 2:**
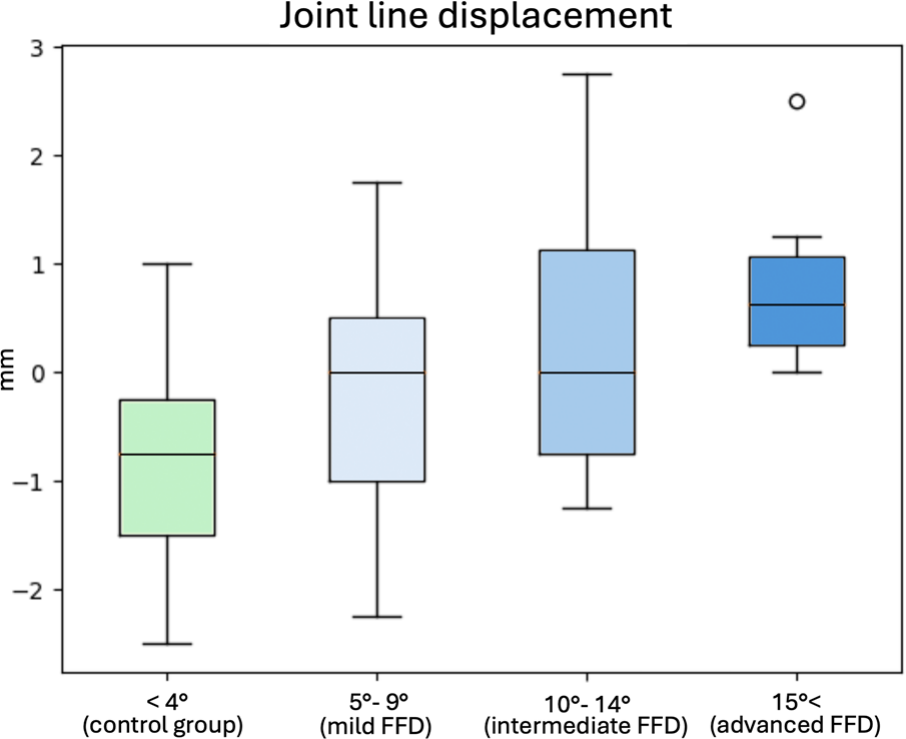
Graphic representation of joint line displacement results for the control group and fixed flexion deformity (FFD) subgroups following joint replacement. Negative values correspond to a lowering of the joint line, while positive values indicate joint line elevation.

The JL was proximally displaced on average by 0.1 ± 1.2 mm (95% CI: −0.3 to 0.5) in the study group and distalised by 0.75 ± 0.9 mm (95% CI: −1.1 to −0.4) in the control group. The average difference between the control and study groups was <1 mm and statistically not significant. Subgroup analysis revealed a lowering of the JL by 0.3 ± 1.2 mm (95% CI: −1 to 0.4) in the mild deformity subgroup and a rise of 0.08 ± 1.3 mm (95% CI: −0.7 to 0.9) and 0.8 ± 0.8 mm (95% CI: 0.1–1.5) in the intermediate and advanced FFD subgroups, respectively (Table A1, Figure 2). The average difference between the control group and any FFD subgroup was within 1.6 ± 1.2 mm, with no statistically significant differences observed.

**Figure 3:**
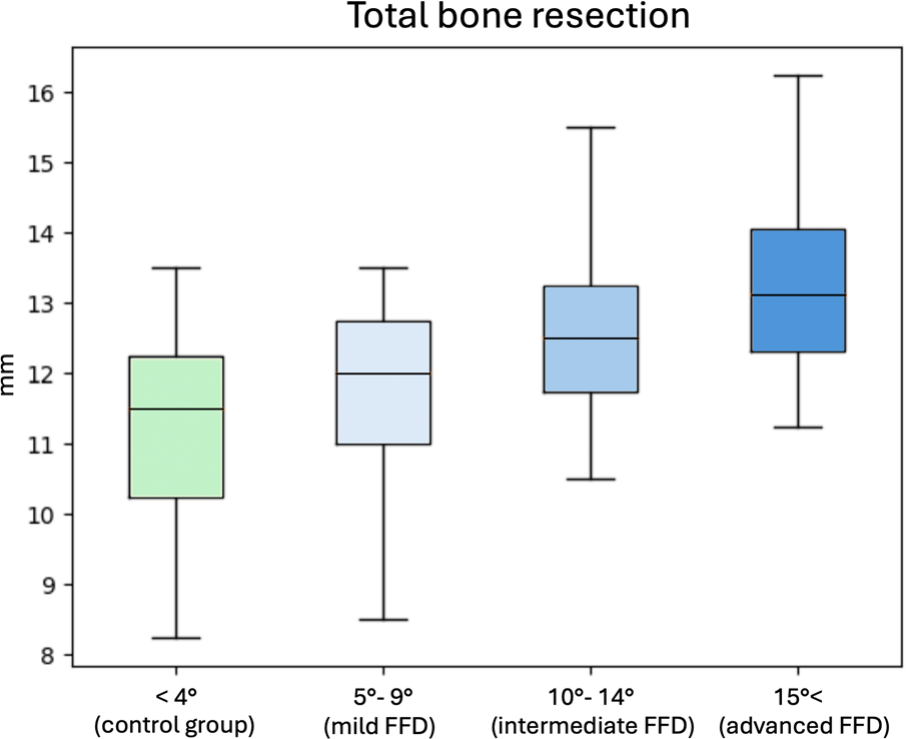
Graphic representation of control group and fixed flexion deformity (FFD) subgroups results of total bone resection after the joint replacement.

The combined thickness of tibial proximal and femoral distal bone cuts measured 12.3 ± 1.6 mm (95% CI: 11.8–12.9) in the study group and 11.4 ± 1.4 mm (95% CI: 10.8–11.9) in the control group. The average difference between the two groups was 0.96 ± 2 mm with no statistical significance. Subgroup assessment demonstrated a proportional increase in bone cut thickness as the extensor deficit increased, with mean measurements ranging from 11.7 ± 1.3 mm in the mild subgroup to 13.3 ± 1.6 mm in the advanced deformity subgroup (Table A1, Figure 3). The average difference between the control and mild‐intermediate groups was <1 mm and statistically not significant. On the other hand, statistical significance was observed between the average bone cut thicknesses in the control and advanced FFD, respectively (Table A1). The single assessment of mean distal femoral bone resection increased from 5.7 ± 0.9 mm (95% CI: 5.4–6.1) in the control group to 7.3 ± 0.8 (95% CI: 6.6–7.9) in the advanced FFD subgroup, while proximal tibial bone resection increased from 5.6 ± 1.1 (95% CI: 5.2–6.0) in the control group to 6 ± 1.8 (95% CI: 4.4–7.5) in the advanced deformity subgroup (Table A1).

## DISCUSSION

This study assessed JL displacement and the amount of bone resected during RA TKA using an FA philosophy, comparing knees with varying degrees of preoperative flexion deformity to those of a control group with complete extension. Our findings demonstrate that this approach effectively corrects FFD while minimising the need for extensive soft tissue releases and distal femoral recuts. It consistently achieved excellent outcomes across different FFD severities, with an average JL elevation of less than 1 mm. Additionally, post‐operative ROM and JL differences showed no statistically significant variations across all subgroups (Table A1).

A deformity in flexion can significantly impact the quality of life by causing increased anterior knee pain, diminished joint function, and greater energy expenditure for activities such as walking and standing [12, 34]. Proper intraoperative management is, therefore, crucial to enhance patient satisfaction and outcomes after knee replacement. Despite this, up to 17% of patients may experience residual flexion contracture post‐surgery [26]. Although this residual flexion rarely increases pain, it can significantly affect daily activities and reduce overall patient satisfaction, as reflected in lower patient‐reported outcomes [22, 51].

Several surgical approaches have been proposed to correct FFD [2]. Most methods involve osteophyte removal, distal femoral recut, and progressive capsular and ligamentous releases up to tenotomies [4]. Among these surgical gestures, distal femoral re‐resection, performed from 2 mm to over 8 mm, has been widely standardised as several studies have shown that each 2 mm of distal femoral resection yields about 4° (3.4–4.8°) of improved knee extension [8, 25, 46, 47]. However, the elevation of the JL provided by these recuts can disturb knee kinematics, resulting in mid‐flexion instability, decreased ROM, patellofemoral complications, and reduced femoral bone stock. About 2 and 4 mm of re‐resection results in 3.1° (64% increase) and 6.5° (111% increase) of mid‐flexion coronal laxity, respectively [6, 29]. Symptoms of altered mid‐flexion include pain, instability during walking, giving way, synovitis and rarely, recurrent hemarthrosis [43]. Severe issues arise with an 8 mm or superior resection, described for flexion deformities greater than 20° [30]. Re‐resection can change the patella's position, impacting the quadriceps muscle lever arm and contact area. This change may lead to patellar tilt, maltracking, or subluxation in susceptible individuals [23, 53]. Moreover, distal femoral epiphysis is vital in revision knee arthroplasty as it provides essential support for the implant, helping to prevent pain and biomechanical issues [38]. Further, soft tissue releases during knee surgery can impair joint function by affecting proprioception and balance, which may lead to reduced patient satisfaction and prolonged recovery time [3, 11]. Soft tissue release may also lengthen the surgical duration, introducing further risks and complications [18, 41, 52].

Because of these numerous issues, adopting a technology‐driven treatment can be highly effective. Robotic devices provide several key benefits, including enhanced deformity assessment, planning and execution. Navigation systems integrated within the RA offer a superior evaluation of flexion deficits compared to traditional goniometers, especially in obese patients, and create a close virtual model of the patient's knee joint [39]. These systems enable meticulous planning for component placement and bone cuts with an error margin of just 1 mm [27, 31, 45].

RA has proved to restore the JL more accurately than both computer‐assisted systems and traditional manual approaches. Both imageless and image‐based systems have demonstrated excellent results, achieving significantly greater accuracy in restoring the non‐pathological JL compared to conventional methods [1, 9, 37].

Particularly, systems endowed with haptic feedback and robotic arms minimise the need for recuts, thereby preserving bone stock and reducing damage to surrounding soft tissues [20].

Robotic surgery has recently been described as managing FFD in two papers on restricted and unrestricted kinematic‐aligned TKA [32, 42]. Both works demonstrated excellent results, correcting full extension within 5° at closure without requiring additional femoral bone resection. However, the KA alignment philosophy, particularly when unrestricted, can lead to excessive internal rotation of the femur, increasing the risk of notching, maltracking and patellar tilt [21, 40].

The FA alignment addresses these limitations, taking advantage of the robotic system's ability to evaluate ligament laxity and customise bone resections in such a way that balanced gaps can be obtained if targeted [36, 44]. This approach can restore both native limb alignment and the JL's height and obliquity reproducibly while addressing significant coronal deformities [7, 49, 50]. Collecting structural data from bony landmarks, soft tissues, and knee kinematics, FA enhances the precision of knee balancing, minimising the need for ligament releases. Moreover, within the balance, asymmetric gaps with 1–2 mm of extra laxity in the lateral compartment are created by adjusting bone resections to replicate natural knee movement [10]. These gaps promote a natural femoral rollback, allowing knee flexion without overloading the femorotibial joint [14, 16, 33]. Along with avoiding ligament releases, asymmetric gaps contribute to more natural knee functionality and higher patient satisfaction [13, 44]. A recent study found that FA achieved a significantly better Forgotten Joint Score at 1‐year post‐surgery compared to restricted KA [17].

In our study, RA combined with FA effectively preserved JL elevation by utilising the system's precise quantitative abilities to optimise flexion‐extension balance regardless of the severity of the flexion deformity. The RA approach allowed for tailor adjustments of component position, including reducing the tibial slope angle, distalising the tibial platform and posteriorising and flexing the femoral component. For deformities exceeding 15°, with a tight extension gap, selecting a CR implant preserving the PCL was preferred. Maintaining the PCL preserves a narrow space in flexion [19]. Therefore, we achieved balanced extension and flexion gaps by increasing the tibial resection only. In cases of PCL insufficiency, we narrowed the flexion gap by upsizing and posteriorising the femoral PS shield, followed by wider tibial resection. These methods ensure effective knee balance and preserve both the JL and the femoral bone stock.

The results of this study reinforce our confidence in this technology. RA and FA are effective for managing flexion and coronal deformities, offering a reliable alternative to the issues encountered with manual instrumentation.

The study acknowledges notable limitations: its retrospective design, the absence of a control group with manual instrumentation and the lack of measurements for preoperative and post‐operative JL height. Furthermore, using both PS and CR inserts reduces the cohort homogeneity; nonetheless, a single radius implant with an 8.5 mm condylar thickness and flexion space balancing was always controlled by optimising the tibial slope. Analysing only the resected epiphyseal thicknesses may produce inaccurate results in cases of significant bone wear. Moreover, the soft tissue balancing procedure is not standardised, and the force applied to the ligaments to balance the knee was not measured, relying on the surgeon's discretion. Finally, the study did not collect data on mediolateral knee stability post‐implant placement or assess functional outcomes. Therefore, more high‐quality research is needed to confirm these results.

## CONCLUSIONS

RA adoption and FA in TKA can effectively correct FFD, minimising bone cuts while preserving anatomical JL level. The average elevation of the JL in the study group was less than 0.5 mm, indicating a reduced risk of mid‐flexion instability when performing FA in patients with fixed‐flexion deformities, even in cases of advanced deformity. Comparing the combined tibial and femoral bone cuts between the study and control groups showed an average difference of 0.95 mm. The total bone loss in the study group was consistently below 2 mm compared to the control group, across all pathological groups. These findings suggest that improved accuracy and precise component positioning in the image‐based interface can significantly enhance balancing, even in advanced FFD cases.

## AUTHOR CONTRIBUTIONS

*Conceptualization*: Alessandro Todesca, George Mihai Avram and Giacomo Pacchiarotti. *Methodology*: Giacomo Pacchiarotti and George Mihai Avram. *Formal analysis*: Giacomo Pacchiarotti and Giovanni Longo. *Investigation*: Giacomo Pacchiarotti. *Writing—original draft preparation*: Giovanni Longo and Giacomo Pacchiarotti. *Writing—review and editing*: Giacomo Pacchiarotti, George Mihai Avram, Domenico Paolicelli and Alessandro Todesca. *Supervision*: Stefano Gumina. All authors have read and agreed to the published version of the manuscript.

## CONFLICT OF INTEREST STATEMENT

The author, Alessandro Todesca, reports consultancy and speaking fees from Stryker. The remaining authors declare no conflicts of interest.

## ETHICS STATEMENT

Given the retrospective nature of the study, which was conducted on already available data and without directly involving human subjects, ethics approval was not necessary. All procedures and analyses were performed in accordance with the ethical standards of the institutional research committee and with the 1964 Helsinki Declaration and its later amendments.

## Supporting information

Supporting information.

## Data Availability

The data that support the findings of this study are available on request from the corresponding author. The data are not publicly available due to privacy or ethical restrictions.

## Appendix

**Table A1:** Analysis of perioperative outcomes and resection results across patients with mild, intermediate and advanced fixed flexion deformity and comparison with the control group.

	Control group, *N* = 56	Mild (5–9°), *N* = 25	*p* a	Intermediate (10–14°), *N* = 24	*p* a	Advanced (>15°), *N* = 15	*p* a
Distal femoral resection (mm)	5.75 ± 0.87	6.19 ± 1.21	0.58	6.58 ± 1.28	0.42	7.31 ± 0.80	0.07
Proximal tibial resection (mm)	5.63 ± 1.07	5.56 ± 1.09	0.79	5.77 ± 0.82	0.78	5.97 ± 1.81	0.91
Total bone resected (mm)	11.38 ± 1.39	11.75 ± 1.34	0.17	12.35 ± 1.57	0.07	13.28 ± 1.58	0.05*
JL displacement (mm)	−0.75 ± 0.86	−0.31 ± 1.21	0.67	0.08 ± 1.28	0.31	0.81 ± 0.80	0.25
Preoperative							
Extension deficit (°)	1.59 ± 2.10	6.92 ± 1.16	0.009*	11.58 ± 1.31	0.011*	18.38 ± 2.20	0.03*
Maximum flexion (°)	125.077 ± 8.75	127 ± 5.61	0.88	125.58 ± 8.08	0.97	117.13 ± 9.26	0.48
Range of motion (°)	123.46 ± 8.30	120.08 ± 6.29	0.54	114 ± 8.28	0.28	98.75 ± 10.62	0.17
HKA angle (°)	179.19 ± 7.41	174.85 ± 6.41	0.50	173.25 ± 6.80	0.44	173.63 ± 5.73	0.49
Post‐operative							
Extension deficit (°)	−0.92 ± 1.90	−0.54 ± 3.15	0.98	−0.33 ± 1.30	0.61	0 ± 1.29	0.21
Maximum flexion (°)	134 ± 4.66	133.23 ± 3.49	0.88	134.5 ± 5.05	0.90	134.43 ± 5.35	0.92
Range of motion (°)	134.92 ± 4.80	133.77 ± 5.07	0.87	134.83 ± 5.72	0.93	134.43 ± 5.32	0.93
HKA angle (°)	178.95 ± 2.39	177.77 ± 1.70	0.46	178.33 ± 2.02	0.66	177 ± 2.88	0.48
mLDFA (°)	90.42 ± 1,73	90.47 ± 1.39	0.79	90.62 ± 1.62	0.81	92 ± 2.33	0.23
mMPTA (°)	89.61 ± 0.99	89.08 ± 0.95	0.57	89.5 ± 0.67	0.78	89.25 ± 1.16	0.79

*Note*: Data distribution is presented as mean values±standard deviation and range.

Abbreviations: HKA, hip–knee–ankle; JL, joint line; mLDFA, mechanical lateral distal femoral angle; mMPTA, mechanical medial proximal tibial angle.

^a^ Differences between each subgroup and control group before and after knee replacement (Mann–Whitney *U* test).

* Significant *p* value (<0.05).

## References

[1] Agrawal VO , Gadekar AP , Vaidya N . Does robotic technology successfully restore the joint line after total knee arthroplasty? A retrospective analysis. Arthroplasty. 2022;4(1):6. 35236508 10.1186/s42836-021-00103-6 PMC8796510

[2] An VVG , Scholes CJ , Fritsch BA . Factors affecting the incidence and management of fixed flexion deformity in total knee arthroplasty: a systematic review. Knee. 2018;25(3):352–359. 29681527 10.1016/j.knee.2018.03.008

[3] Attfield SF , Wilton TJ , Pratt DJ , Sambatakakis A . Soft‐tissue balance and recovery of proprioception after total knee replacement. J Bone Joint Surg Br. 1996;78(4):540–545. 8682816

[4] Bellemans J , Vandenneucker H , Victor J , Vanlauwe J . Flexion contracture in total knee arthroplasty. Clin Orthop Relat Res. 2006;452:78–82. 16924174 10.1097/01.blo.0000238791.36725.c5

[5] Campbell TM , McGonagle D . Flexion contracture is a risk factor for knee osteoarthritis incidence, progression and earlier arthroplasty: data from the Osteoarthritis Initiative. Ann Phys Rehabil Med. 2021;64(2):101439. 33065299 10.1016/j.rehab.2020.09.005

[6] Chalmers BP , Elmasry SS , Kahlenberg CA , Mayman DJ , Wright TM , Westrich GH , et al. Additional distal femoral resection increases mid‐flexion coronal laxity in posterior‐stabilized total knee arthroplasty with flexion contracture: a computational study. Bone Joint J. 2021;103–B(6 Suppl A):87–93. 10.1302/0301-620X.103B6.BJJ-2020-2444.R1 34053287

[7] Clark G , Steer R , Wood D . Functional alignment achieves a more balanced total knee arthroplasty than either mechanical alignment or kinematic alignment prior to soft tissue releases. Knee Surg Sports Traumatol Arthrosc. 2023;31(4):1420–1426. 36116071 10.1007/s00167-022-07156-3 PMC10050049

[8] Cross MB , Nam D , Plaskos C , Sherman SL , Lyman S , Pearle AD , et al. Recutting the distal femur to increase maximal knee extension during TKA causes coronal plane laxity in mid‐flexion. Knee. 2012;19(6):875–879. 22727760 10.1016/j.knee.2012.05.007

[9] Ensini A , Catani F , Biasca N , Belvedere C , Giannini S , Leardini A . Joint line is well restored when navigation surgery is performed for total knee arthroplasty. Knee Surg Sports Traumatol Arthrosc. 2012;20(3):495–502. 21625830 10.1007/s00167-011-1558-1

[10] Ferle M , Guo R , Hurschler C . The laxity of the native knee: a meta‐analysis of in vitro studies. J Bone Joint Surg. 2019;101(12):1119–1131. 31220029 10.2106/JBJS.18.00754

[11] Gu Y , Roth JD , Howell SM , Hull ML . How frequently do four methods for mechanically aligning a total knee arthroplasty cause collateral ligament imbalance and change alignment from normal in white patients? AAOS exhibit selection. J Bone Joint Surg. 2014;96(12):e101. 24951744 10.2106/JBJS.M.00306

[12] Harato K , Nagura T , Matsumoto H , Otani T , Toyama Y , Suda Y . Extension limitation in standing affects weight‐bearing asymmetry after unilateral total knee arthroplasty. J Arthroplasty. 2010;25(2):225–229. 19264442 10.1016/j.arth.2009.02.003

[13] Hazratwala K , Gouk C , Wilkinson MPR , O'Callaghan WB . Navigated functional alignment total knee arthroplasty achieves reliable, reproducible and accurate results with high patient satisfaction. Knee Surg Sports Traumatol Arthrosc. 2023;31(9):3861–3870. 36917248 10.1007/s00167-023-07327-w PMC10435654

[14] Hill PF , Vedi V , Williams A , Iwaki H , Pinskerova V , Freeman MAR . Tibiofemoral movement 2: the loaded and unloaded living knee studied by MRI. J Bone Joint Surg Br. 2000;82(8):1196–1198. 11132286 10.1302/0301-620x.82b8.10716

[15] Insall JN , Dorr LD , Scott RD , Scott WN . Rationale of the knee society clinical rating system. Clin Orthop Relat Res. 1989;(248):13–14. 2805470

[16] Iwaki H , Pinskerova V , Freeman MAR . Tibiofemoral movement 1: the shapes and relative movements of the femur and tibia in the unloaded cadaver knee. J Bone Joint Surg Br. 2000;82(8):1189–1195. 11132285 10.1302/0301-620x.82b8.10717

[17] Kafelov M , Batailler C , Shatrov J , Al‐Jufaili J , Farhat J , Servien E , et al. Functional positioning principles for image‐based robotic‐assisted TKA achieved a higher Forgotten Joint Score at 1 year compared to conventional TKA with restricted kinematic alignment. Knee Surg Sports Traumatol Arthrosc. 2023;31(12):5591–5602. 37851026 10.1007/s00167-023-07609-3

[18] Kang J , Jiang X , Wu B . Analysis of risk factors for lower‐limb deep venous thrombosis in old patients after knee arthroplasty. Chin Med J. 2015;128(10):1358–1362. 25963358 10.4103/0366-6999.156782 PMC4830317

[19] Kayani B , Konan S , Horriat S , Ibrahim MS , Haddad FS . Posterior cruciate ligament resection in total knee arthroplasty: the effect on flexion‐extension gaps, mediolateral laxity, and fixed flexion deformity. Bone Joint J. 2019;101–B(10):1230–1237. 10.1302/0301-620X.101B10.BJJ-2018-1428.R2 31564152

[20] Kayani B , Konan S , Pietrzak JRT , Haddad FS . Iatrogenic bone and soft tissue trauma in robotic‐arm assisted total knee arthroplasty compared with conventional jig‐based total knee arthroplasty: a prospective cohort study and validation of a new classification system. J Arthroplasty. 2018;33(8):2496–2501. 29699827 10.1016/j.arth.2018.03.042

[21] Koh DTS , Woo YL , Yew AKS , Yeo SJ . Kinematic aligned femoral rotation leads to greater patella tilt but similar clinical outcomes when compared to traditional femoral component rotation in total knee arthroplasty. A propensity score matched study. Knee Surg Sports Traumatol Arthrosc. 2021;29(4):1059–1066. 32488370 10.1007/s00167-020-06081-7

[22] Koh IJ , Chang CB , Kang YG , Seong SC , Kim TK . Incidence, predictors, and effects of residual flexion contracture on clinical outcomes of total knee arthroplasty. J Arthroplasty. 2013;28(4):585–590. 23142447 10.1016/j.arth.2012.07.014

[23] König C , Sharenkov A , Matziolis G , Taylor WR , Perka C , Duda GN , et al. Joint line elevation in revision TKA leads to increased patellofemoral contact forces. J Orthop Res. 2010;28(1):1–5. 19637213 10.1002/jor.20952

[24] Li G , Park SE , DeFrate LE , Schutzer ME , Ji L , Gill TJ , et al. The cartilage thickness distribution in the tibiofemoral joint and its correlation with cartilage‐to‐cartilage contact. Clin Biomech. 2005;20(7):736–744. 10.1016/j.clinbiomech.2005.04.001 15963613

[25] Liu DW , Reidy JF , Beller EM . The effect of distal femoral resection on fixed flexion deformity in total knee arthroplasty. J Arthroplasty. 2016;31(1):98–102. 26321077 10.1016/j.arth.2015.07.033

[26] Luo CF . Reference axes for reconstruction of the knee. Knee. 2004;11(4):251–257. 15261208 10.1016/j.knee.2004.03.003

[27] Lustig S , Fleury C , Goy D , Neyret P , Donell ST . The accuracy of acquisition of an imageless computer‐assisted system and its implication for knee arthroplasty. Knee. 2011;18(1):15–20. 20060724 10.1016/j.knee.2009.12.010

[28] Lustig S , Sappey‐Marinier E , Fary C , Servien E , Parratte S , Batailler C . Personalized alignment in total knee arthroplasty: current concepts. SICOT‐J. 2021;7:19. 33812467 10.1051/sicotj/2021021 PMC8019550

[29] Luyckx T , Vandenneucker H , Ing LS , Vereecke E , Ing AV , Victor J . Raising the joint line in TKA is associated with mid‐flexion laxity: a study in cadaver knees. Clin Orthop Relat Res. 2018;476(3):601–611. 29443845 10.1007/s11999.0000000000000067 PMC6260050

[30] Matziolis G , Loos M , Böhle S , Schwerdt C , Roehner E , Heinecke M . Effect of additional distal femoral resection on flexion deformity in posterior‐stabilized total knee arthroplasty. Knee Surg Sports Traumatol Arthrosc. 2020;28(9):2924–2929. 31420688 10.1007/s00167-019-05675-0

[31] Miyasaka T , Kurosaka D , Saito M , Omori T , Ikeda R , Marumo K . Accuracy of computed tomography‐based navigation‐assisted total knee arthroplasty: outlier analysis. J Arthroplasty. 2017;32(1):47–52. 27369304 10.1016/j.arth.2016.05.069

[32] Moya‐Angeler J , León‐Muñoz VJ , Jimenez‐Soto C , Huber K , Christen B , Calliess T . Fixed flexion contracture can successfully be addressed with exact preservation of the femoral joint line and only minimal increase of tibia resection in the concept of kinematically aligned total knee arthroplasty. J Pers Med. 2023;13(5):868. 37241038 10.3390/jpm13050868 PMC10223136

[33] Murphy GT , Shatrov J , Duong J , Fritsch BA . How does the use of quantified gap‐balancing affect component positioning and limb alignment in robotic total knee arthroplasty using functional alignment philosophy? A comparison of two robotic platforms. Int Orthop. 2023;47(5):1221–1232. 36740610 10.1007/s00264-022-05681-x PMC10079723

[34] Murphy MT , Skinner TL , Cresswell AG , Crawford RW , Journeaux SF , Russell TG . The effect of knee flexion contracture following total knee arthroplasty on the energy cost of walking. J Arthroplasty. 2014;29(1):85–89. 23725927 10.1016/j.arth.2013.04.039

[35] Oussedik S , Abdel MP , Victor J , Pagnano MW , Haddad FS . Alignment in total knee arthroplasty. Bone Joint J. 2020;102–B(3):276–279. 10.1302/0301-620X.102B3.BJJ-2019-1729 32114811

[36] Parratte S , Van Overschelde P , Bandi M , Ozturk BY , Batailler C . An anatomo‐functional implant positioning technique with robotic assistance for primary TKA allows the restoration of the native knee alignment and a natural functional ligament pattern, with a faster recovery at 6 months compared to an adjusted mechanical technique. Knee Surg Sports Traumatol Arthrosc. 2023;31(4):1334–1346. 35552475 10.1007/s00167-022-06995-4

[37] Popat R , Albelooshi A , Mahapatra P , Bollars P , Ettinger M , Jennings S , et al. Improved joint line and posterior offset restoration in primary total knee replacement using a robotic‐assisted surgical technique: an international multi‐centre retrospective analysis of matched cohorts. PLoS One. 2022;17(8):e0272722. 36006969 10.1371/journal.pone.0272722 PMC9409519

[38] Porteous AJ , Hassaballa MA , Newman JH . Does the joint line matter in revision total knee replacement? J Bone Joint Surg Br. 2008;90–B(7):879–884. 10.1302/0301-620X.90B7.20566 18591596

[39] Ritter MA , Lutgring JD , Davis KE , Berend ME , Pierson JL , Meneghini RM . The role of flexion contracture on outcomes in primary total knee arthroplasty. J Arthroplasty. 2007;22(8):1092–1096. 18078875 10.1016/j.arth.2006.11.009

[40] Rivière C , Iranpour F , Harris S , Auvinet E , Aframian A , Parratte S , et al. Differences in trochlear parameters between native and prosthetic kinematically or mechanically aligned knees. Orthop Traumatol Surg Res. 2018;104(2):165–170. 29223778 10.1016/j.otsr.2017.10.009

[41] Rivière C , Villet L , Jeremic D , Vendittoli PA . What you need to know about kinematic alignment for total knee arthroplasty. Orthop Traumatol Surg Res. 2021;107(1S):102773. 33333274 10.1016/j.otsr.2020.102773

[42] Sappey‐Marinier E , Bini S . Unrestricted kinematic alignment corrects fixed flexion contracture in robotically aligned total knees without raising the joint line in extension. J Exp Orthop. 2023;10(1):114. 37950808 10.1186/s40634-023-00670-4 PMC10640542

[43] Schroer WC , Berend KR , Lombardi AV , Barnes CL , Bolognesi MP , Berend ME , et al. Why are total knees failing today? Etiology of total knee revision in 2010 and 2011. J Arthroplasty. 2013;28(8 Suppl):116–119. 23954423 10.1016/j.arth.2013.04.056

[44] Shatrov J , Batailler C , Sappey‐Marinier E , Gunst S , Servien E , Lustig S . Functional alignment philosophy in total knee arthroplasty—rationale and technique for the varus morphotype using a CT based robotic platform and individualized planning. SICOT‐J. 2022;8:11. 35363136 10.1051/sicotj/2022010 PMC8973302

[45] Sires JD , Craik JD , Wilson CJ . Accuracy of bone resection in MAKO total knee robotic‐assisted surgery. J Knee Surg. 2021;34(7):745–748. 31694057 10.1055/s-0039-1700570

[46] Smith CK , Chen JA , Howell SM , Hull ML . An in vivo study of the effect of distal femoral resection on passive knee extension. J Arthroplasty. 2010;25(7):1137–1142. 19643566 10.1016/j.arth.2009.05.030

[47] Su EP . Fixed flexion deformity and total knee arthroplasty. J Bone Joint Surg Br. 2012;94(11 Suppl A):112–115. 23118396 10.1302/0301-620X.94B11.30512

[48] Sun H , Zhang H , Wang T , Zheng K , Zhang W , Li W , et al. Biomechanical and finite‐element analysis of femoral pin‐site fractures following navigation‐assisted total knee arthroplasty. J Bone Joint Surg. 2022;104(19):1738–1749. 36197326 10.2106/JBJS.21.01496

[49] Tuecking LR , Savov P , Zander M , Jeremic D , Windhagen H , Ettinger M . Comparable accuracy of femoral joint line reconstruction in different kinematic and functional alignment techniques. Knee Surg Sports Traumatol Arthrosc. 2023;31(9):3871–3879. 36917247 10.1007/s00167-023-07360-9

[50] Vaidya NV , Deshpande AN , Panjwani T , Patil R , Jaysingani T , Patil P . Robotic‐assisted TKA leads to a better prosthesis alignment and a better joint line restoration as compared to conventional TKA: a prospective randomized controlled trial. Knee Surg Sports Traumatol Arthrosc. 2022;30(2):621–626. 33165631 10.1007/s00167-020-06353-2

[51] Van Onsem S , Van Der Straeten C , Arnout N , Deprez P , Van Damme G , Victor J . A new prediction model for patient satisfaction after total knee arthroplasty. J Arthroplasty. 2016;31(12):2660–2667.e1. 27506723 10.1016/j.arth.2016.06.004

[52] Yoon JR , Han SB , Jee MK , Shin YS . Comparison of kinematic and mechanical alignment techniques in primary total knee arthroplasty: a meta‐analysis. Medicine. 2017;96(39):e8157. 28953661 10.1097/MD.0000000000008157 PMC5626304

[53] Yoshii I , Whiteside LA , White SE , Milliano MT . Influence of prosthetic joint line position on knee kinematics and patellar position. J Arthroplasty. 1991;6(2):169–177. 1875209 10.1016/s0883-5403(11)80013-6

